# Hyperprogression to a dual immune blockade followed by subsequent response with cabozantinib in metastatic poor-risk clear cell renal cell carcinoma with NOTCH mutation

**DOI:** 10.18632/oncotarget.27598

**Published:** 2020-06-02

**Authors:** Javier Molina-Cerrillo, Teresa Alonso-Gordoa, Alfredo Carrato, Enrique Grande

**Affiliations:** ^1^ Medical Oncology Department, Ramón y Cajal University Hospital, Madrid, Spain; ^2^ MD Anderson Cancer Center, Madrid, Spain

**Keywords:** immunotherapy, renal cell carninoma, cabozantinib, NOTCH

## Abstract

Currently, more and more patients receive first-line treatment with immunotherapy combinations and not all patients respond in metastatic renal cell carcinoma. After IO-IO progression, we don’t have a standard of treatment because it is not available prospective data on this setting. We present the case of a patient with metastatic renal cell carcinoma who suffered hyperprogression with IO-IO combination in first line. Second line with cabozantinib results in a deep response of the disease. We performed a Foundation One testing to the patient which showed a mutation in NOTCH. The molecular mechanism to explain patient’s response, it’s the probably crosstalk between MET and NOTCH pathway. Nowadays, there is not clear the subsequent treatment in those patients who progress to IO-IO first line. More efforts in biomarkers development should be made to better selection of patients treatment along the disease.

## INTRODUCTION

We present the case of a 50-year-old male who came to the emergency room in March 2019 due to the recent appearance of a mass in the lower region of the left thigh.

Magnetic resonance was performed and described a 4.5 × 4 cm lesion in the thickness of the vast intermediate muscle that was suspicious of a primary tumor of the soft tissue vs metastases from another origin. Body CT scan revealed the presence of myocardial implants with extension towards the left ventricular lumen measuring up to 50 mm and pericardial implants, micronodules in both lungs, several lesions in the right gluteus, up to 21 mm and a mass in the upper pole of the left kidney of 6.8 × 5.5 cm.

The case was discussed at the genitourinary tumor board and decided to perform a biopsy of both the mass in the thickness of the intermediate layer and the kidney. The pathological results showed that both lesions were affected by a clear cell carcinoma (ccRCC), Fuhrman grade IV.

On physical examination, the patient presented a mass in the left thigh region and Karnofsky Index (KI) was 90%. The blood test showed: Hemoglobin 11.7 gr/dl (Reference[Ref]: 13–17.5 gr/dL), Neutrophils 9,450/μL (Ref: 1,500–7,700/μL), Platelets 142,000/μL (Ref: 140–400,000/μL), LDH 412 U/L (Ref: 140–240 U/L), Calcium 9.2 mg/dL (Ref: 8.7–10.3 mg/dL)

Considering the final diagnosis as a poor prognosis clear cell renal cell carcinoma stage IV based on the MSKCC and IMDC prognostic criteria, the patient was offered to receive first-line treatment with Nivolumab 3 mg/Kg and Ipilimumab 1 mg/Kg every 3 weeks in the induction phase in April 2019 based on Checkmate 214 results [1].

After 2 cycles, in May 2019, the patient came to the clinic showing a significant clinical deterioration with a KI of 50%. In the physical examination, the patient presented new painful and palpable masses in the left dorsolumbar region and right gluteus. The radiological assessment confirmed the clinical suspicious of disease progression with an increase in the renal tumor mass of 7.7 × 5.9 cm, new intraabdominal implants in the right perirenal space of 4 cm and left of 3 cm, right hypochondrium of 5.5 cm and left iliac fossa of 4 cm. New implants were also identified within the soft tissue at different levels: retropectoral, axillary, paravertebral, gluteus and psoas with a diameter between 3.8 cm to 4.5 cm. In addition, bilateral lung nodules of new appearance were reported, being the largest ones of 6.5 mm in left superior lobe, as well as disease progression at myocardial and pericardial sites.

**Figure 1:**
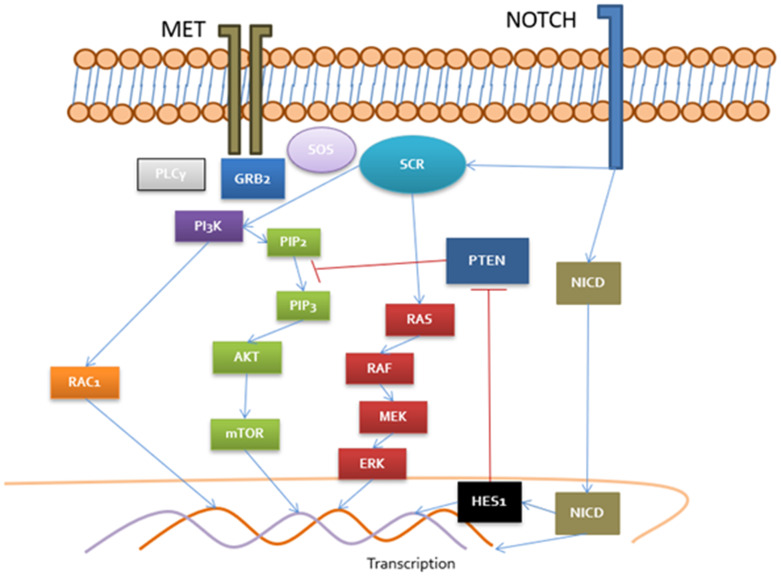
Cross-talk between MET/NOTCH pathways MET recruit PLCγ, GRB2 and SOS which activate DAG receptors (PKC), RAS/RAF/MEK, PI3K/RAC and Akt/mTOR. PI3K pathway are negatively regulated by PTEN. NOTCH upregulate HES1 transcriptional factor implied in negative control of PTEN. Moreover, SCR in a central pathway in MET and NOTCH activation consisting in a key regulator in both signalization routes. NOTCH is capable to control MET promoter and MET regulate expression of delta promoter (NOTCH ligand) in nucleus.

According to this situation of hyperprogression, we considered performing a foundation one in primary tumor sample achieving mutations in VHL (L158fs*14), NOTCH2(R65*), BAP1(P488fs*82). MSI-stable, low mutational tumor burden. Considering the mutation in NOTCH and its possible relationship with HGF/MET pathway [2, 3] (Figure 1) and knowing that the alterations in BAP1 are related with worse prognosis, we decided to offer the patient a second-line treatment with cabozantinib 60 mg daily. The patient began this treatment in early June 2019 with close monitoring of adverse events and tumor assessment. After 2 weeks of treatment, the patient began to experience significant clinical improvement, recovering to a KI of 90% and with an objective tumor reduction of the visible implants in the left dorsolumbar region, right gluteus muscle and disappearance in vast intermediate lesion. Treatment with cabozantinib was well tolerated with grade II diarrhea that had an adequate response to loperamide treatment.

The radiological assessment performed in July 2019 showed that most of the soft tissue implants had disappeared and only some of them continued with a significant reduction in size, as well as those in the peritoneum, myocardial, pericardial tissue and lung.

**Figure 2:**
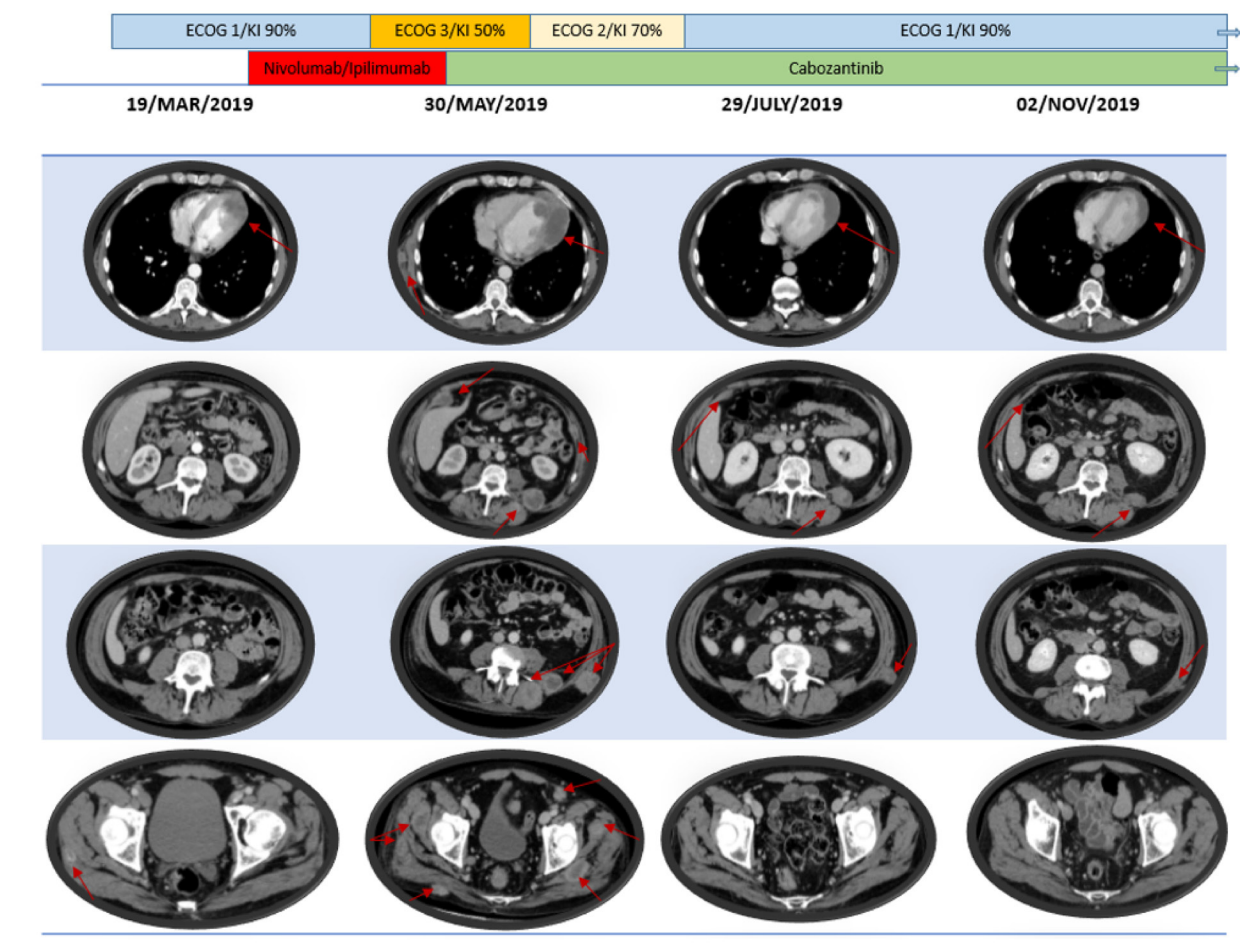
Patient’s radiology and clinical outcome according to systemic treatments.

The patient is currently under treatment with cabozantinib at the same dose level and acceptable tolerability. The last radiological evaluation performed in November 2019 showed the disappearance of the soft tissue lesions, practical resolution of peritoneal implants, no visible lung disease and significant reduction in size of the primary renal tumor mass (Figure 2).

In the last years, phase III clinical trials are including new practice-changing combinations in the first line treatment of advanced kidney cancer, based in double IO (IO-IO) or in combination with antiangiogenics (IO-VEGFR). These combinations are interestingly increasing the complete response rates and survival [4]. However, patients may develop eventual resistance to these combinations or being primary refractory. In pivotal trials, as much as 11% of the patients treated with the combination of IO-VEGFR and 20% when used the dual blockade of IO-IO combinations are showing radiology progression as best response according to RECIST criteria. No prospective data are reported so far after failure to combinations as a first approach in metastatic RCC patients, therefore VEGFR inhibitors are widely used and recommended in main guidelines based on retrospective series.

Given the unequivocal clinical and radiology hyperprogression that this patient presented, a second line treatment that could induce an early response was required. In this setting, we considered that cabozantinib might be the best available option based on its mechanism of action as an inhibitor of VEGFR, mainly type 2, but also of other therapeutic targets involved in molecular mechanisms of tumor progression and resistance, such as Axl and c-MET, taking into account the alteration in NOTCH2 of the patient and its relationship with this molecular pathways and MET promoter [2, 3, 5] and its favorable results in disease control rate obtained in different clinical trials [6].

As far as we know, there are no reported cases of hyperprogression in kidney cancer patients receiving the combination of nivolumab and ipilimumab. In addition, there are few data coming from retrospective series on the use of TKI after progression to previous IO treatment in metastatic renal cancer. Those data show some activity from TKIs in this setting, but in patients not responding to the IO-IO combination, response durations are below 5 months, representing a subgroup of patients with a particularly poor prognosis. This evidence is even more limited in the case of cabozantinib, as only a small percentage of patients included in these retrospective studies received this drug [7, 8]. In this report, the patient is benefiting from an early and profound response of cabozantinib for at least 6 months with an improvement in his quality of life.

In conclusion, there may be hyperprogressions with the new combinations in RCC, the NOTCH pathway may be activated in some patients and MET inhibition may be key to its control. In this case it is demonstrated that a TKI such cabozantinib can control hyperprogression phenomena in 2L of m ccRCC. Clinical trials are currently ongoing in this setting trying to elucidate, prospectively, which is the best strategy [8–10]. In this sense, biomarker research is essential to identify those tumor profiles that provide information about the best approach in the first line setting and beyond.
