# Addendum: Hyperprogression to a dual immune blockade followed by subsequent response with cabozantinib in metastatic poor-risk clear cell renal cell carcinoma with NOTCH mutation

**DOI:** 10.18632/oncotarget.28312

**Published:** 2022-11-17

**Authors:** Javier Molina-Cerrillo, Teresa Alonso-Gordoa, Alfredo Carrato, Enrique Grande

**Affiliations:** ^1^Medical Oncology Department, Ramón y Cajal University Hospital, Madrid, Spain; ^2^MD Anderson Cancer Center, Madrid, Spain

**This article has an addendum:** The patient provided written informed consent.

Original article: Oncotarget. 2020; 11:2137–2140. 2137-2140. https://doi.org/10.18632/oncotarget.27598

